# An integrated Biobank in the Swedish Heart Failure Registry—clinomics, proteomics, transcriptomics and genomics

**DOI:** 10.1093/eschf/xvag092

**Published:** 2026-04-08

**Authors:** Camilla Hage, Therese Andersson, Christina Christersson, Cecilia Linde, Lars H Lund, Patric Karlström, Dina Chatziapostolou, Viveka Dagner, Frida Granström, Anette Gylling, Åsa Jonsson, Pernilla Haglund, Jenny Högberg, Annika Odenstedt, Ulrika Viklund, Martin Magnusson, J Gustav Smith, Barna Szabó-Söderberg, Erik Östgärd Thunström, Ulf Dahlström

**Affiliations:** Department of Medicine, Karolinska Institutet, Stockholm S-171 64, Sweden; Department of Cardiology, Karolinska University Hospital, Stockholm S-171 64, Sweden; Departement of Public Health and Clinical Medicine, Umea University Hospital, UmeåS-901 85 Sweden; Department of Medical Sciences, Cardiology, Uppsala University, Uppsala S-751 85, Sweden; Department of Medicine, Karolinska Institutet, Stockholm S-171 64, Sweden; Department of Cardiology, Karolinska University Hospital, Stockholm S-171 64, Sweden; Department of Medicine, Ryhov Hospital, Jönköping S-551 85, Sweden; Department of Health, Medicine and Caring Sciences, Linköping University, Linköping S-581 91, Sweden; Department of Cardiology, Skåne University Hospital, Malmö S-205 02, Sweden; Department of Cardiology, Clinical Sciences, Lund University and Skåne University Hospital, Lund S-221 85, Sweden; Department of Cardiology, Karolinska University Hospital, Stockholm S-171 64, Sweden; Department of Cardiology and Department of Health, Medicine and Caring Sciences, Linköping University, Linköping S-581 91, Sweden; Department of Medicine, Ryhov Hospital, Jönköping S-551 85, Sweden; Heart-, Lung- and Physiology Clinic, Örebro University Hospital, Örebro S-701 85, Sweden; Department of Cardiology, Karolinska University Hospital, Stockholm S-171 64, Sweden; Department of Medicine, Sahlgrenska University Hospital/Östra Sjukhuset, Göteborg S-416 85, Sweden; Departement of Public Health and Clinical Medicine, Umea University Hospital, UmeåS-901 85 Sweden; Department of Cardiology, Skåne University Hospital, Malmö S-205 02, Sweden; Department of Clinical Sciences, Lund University, Malmö S-221 85, Sweden; Wallenberg Center for Molecular Medicine, Lund University, Lund S-221 85, Sweden; Hypertension in Africa Research Team (HART), North-West University, Potchefstroom, South Africa; Department of Cardiology, Clinical Sciences, Lund University and Skåne University Hospital, Lund S-221 85, Sweden; Wallenberg Center for Molecular Medicine and Lund University Diabetes Center, Lund University, Lund S-221 85, Sweden; Department of Molecular and Clinical Medicine, Institute of Medicine, Gothenburg University and Sahlgrenska University Hospital, Gothenburg S-418 77, Sweden; Science for Life Laboratory, Gothenburg University, Gothenburg S-418 77, Sweden; Heart-, Lung- and Physiology Clinic, Örebro University Hospital, Örebro S-701 85, Sweden; Department of Medicine, Sahlgrenska University Hospital/Östra Sjukhuset, Göteborg S-416 85, Sweden; Department of Molecular and Clinical Medicine, Institute of Medicine, Sahlgrenska Academy, Gothenburg University, Gothenburg S-418 77, Sweden; Department of Cardiology and Department of Health, Medicine and Caring Sciences, Linköping University, Linköping S-581 91, Sweden

**Keywords:** Heart failure, Design, Clinomics, Proteomics, Transcriptomics, Genomics

## Abstract

**Aims:**

To build a comprehensive biobank integrated in the Swedish Heart Failure Registry (SwedeHF) comprising comprehensive clinomic data, proteomic, transcriptomic and genomic information in combination with clinical and diagnostic characteristics and additional ICD-code registry data.

**Methods:**

Blood and urine samples will be biobanked at SwedeHF registration with an optional second sampling after 6 months in patients with HF attending routine clinical visits at nine hospitals with access to healthcare integrated biobanking. Circulating and urine biomarkers will be investigated by proteomic, metabolomic, transcriptomic profiling, explored with genetic data. Sample size assessments were based on the BIOSTAT-CHF cohort and doubled to fulfil all aims targeting 5000 patients.

**Results:**

The first 1348 enrolled patients were median 72 years, 30% females, 65% HFrEF and 11% HFpEF. Median NT-proBNP was 1240 (quartile 1–3; 470–2830) pg/mL. This was comparable to the 8506 patients with an index registration in SwedeHF during 2023 with 52% HFrEF and 20% HFpEF, age 75 years, 36% females and NT-proBNP 1560 [629–3617] pg/mL.

**Conclusions:**

We are building a high-quality detailed biobank linked to SwedeHF, the world’s largest continuous HF registry, consisting of plasma, serum, whole blood and urine samples. The Biobank will enable studies exploring underlying disease mechanisms in HF and response to HF treatment, paving the way for precision medicine and novel drug targets. It will also generate a structure for biobanking in Registry-based Randomized Controlled Trials within the national SwedeHF registry.

**ClinicalTrials.gov Identifier:**

NCT06435585

## Introduction

Heart failure (HF) affects 2%–3% of the Western population, with increasing prevalence due to the increased longevity and aging of the population.^1^ In Sweden 200 000–300 000 people live with HF, a syndrome associated with poor quality of life and shorter life expectancy. HF is the most common cause of hospitalization over the age of 65 years and mortality rates remain high.^2^ HF is according to Universal Definition and Classification and international guidelines categorized according to the left ventricular ejection fraction (LVEF) as reduced (≤40%; HFrEF), moderately reduced (41%–49%; HFmrEF) or preserved (≥50%; HFpEF).^3^ The most recent category is HF with improved LVEF (HFimpEF; HF with a baseline LVEF ≤40%, a ≥10 point increase from baseline LVEF, and a second measurement of LVEF >40%).^4^

HFrEF patients comprise 40%–60% of the HF population.^1^ Therapies targeting maladaptive neurohormonal activation such as beta-blockers, ACE inhibitors (ACEi), angiotensin receptor-neprilysin inhibitors (ARNi), and mineralocorticoid receptor antagonists (MRAs) added by sodium-glucose co-transporter 2/1 (SGLT2/1) inhibitors, have demonstrated significant improvements in clinical outcomes in randomized controlled trials (RCTs) and are recommended as Guideline Directed Medical Therapy (GDMT).^3^ Still, mortality remains high. Current therapies may be more or less beneficial for certain HF patient groups differing in age, sex, aetiology and more implying insufficient knowledge on underlying mechanisms.

HFmrEF, reported in 14%–24% of HF patients,^1^ shares many features with HFrEF and effects of GDMT studied in HFrEF also seems to extend to HFmrEF.^5^ The beneficial effects have primarily been demonstrated in post-hoc- and meta-analyses and may apply to selected patients or sub-groups.^3^

The HFpEF syndrome is almost as common as HFrEF, reported in 24%–47% of HF populations, and associated with nearly the same rates of morbidity and mortality as HFrEF.^6^ Although specific aetiologies exist (i.e. hypertension, hypertrophic cardiomyopathy, diabetes), many patients do not have a specific cardiac underlying cause. Patients with HFpEF are older with more co-morbidities which may drive the poor outcomes suggesting a greater heterogeneity of phenotypes and/or a different underlying pathophysiology in HFpEF than in HFrEF.^6,7^ The overarching hypothesis is that HFpEF has comorbidity-driven inflammation as a unifying mechanism while HFrEF is primarily driven by the maladaptive compensatory neurohormonal activation.^8–10^ Outcome trials in HFpEF were for a long time unsuccessful in reducing outcomes. Presently GDMT is limited to SGLT2/1 inhibitors,^11^ even if novel MRAs^12^ and glucagon-like peptide-1 agonists^13^ may be options in the future.

Circulating biomarkers have contributed to development of GDMT informing on underlying pathophysiology and identifying modifiable druggable targets. The early studies described neurohormonal activation by increased concentrations of norepinephrine and renin and their association with low cardiac output and deteriorating systolic dysfunction. This initial knowledge has grown and today we have successful pharmacological treatments in HFrEF extending beyond inhibiting the renin-angiotensin and beta-adrenergic systems in form of enhancing endogenous B-type natriuretic peptide (BNP), inhibiting sodium-glucose cotransporters and stimulation of soluble guanylate cyclase.^3^

By using biomarkers, we can further extend knowledge on HF pathophysiology, biological functions and genetic susceptibility. We can study response to HF treatment to understand and utilize existing treatment, use biomarker-guided pharmacologic therapy and develop novel alternatives. Preferably such studies should be conducted in a general non-selected population of HF patients. For this purpose, we have initiated a Biobank as a part of SwedeHF, the national quality HF registry in Sweden. Further, the structure of a Biobank within SwedeHF creates a platform well suited for performing registry-based randomized controlled trials (RRCTs) more pragmatic and simpler to perform than the traditional RCT. Presently one RRCT has been conducted in SwedeHF.^14^ The concept of streamlined research usually does not allow biobanking. The SwedeHF Biobank offer a structure, with data collection and biobanking integrated in clinical care in hospitals all over Sweden, facilitating enrolling nationwide real-world HF patients with biobank sampling in RRCTs. The SwedeHF Biobank was initiated in January 2021, with the ambition to collect peripheral blood and urine from 5000 patients. We herein report the study design and baseline characteristic for the first enrolled 1348 patients.

### Objectives

The overall objective is to build a large, high-quality Biobank with standardized handling of biomaterial within the nationwide SwedeHF registry enrolling patients with chronic and acute for HF regardless LVEF.

1. To identify molecular HF sub-phenotypes sharing pathophysiological mechanisms by comparing the plasma proteome and metabolome using unsupervised clustering approaches, and their association with outcome in patients with HF.
2. To identify novel upstream regulators of HF, pathways and effector mechanisms by comparing the plasma proteome and metabolome, and their association with outcome in patients with HFrEF and HFpEF.
3. To identify polymorphisms and as a long-term goal explore the contribution of rare gene variants with phenotypic impact on outcomes in patients with HFrEF and HFpEF, respectively.
4. To provide opportunities for future research within SwedeHF and structure for future RRCTs including biological material.

### Rationale

The national SwedeHF Biobank will provide detailed clinical information in combination with pathophysiological mechanisms in HF by circulating biomarkers and genetic determinants. Large real-world registry data in combination with molecular information will pave the way for clinomics with precision medicine with individualized treatment and development of novel therapeutic options across LVEF categories in HF. It will also provide unique opportunities for future research within SwedeHF and provide a structure for future research and RRCTs.

## Study design

### Patients

**Figure 1:**
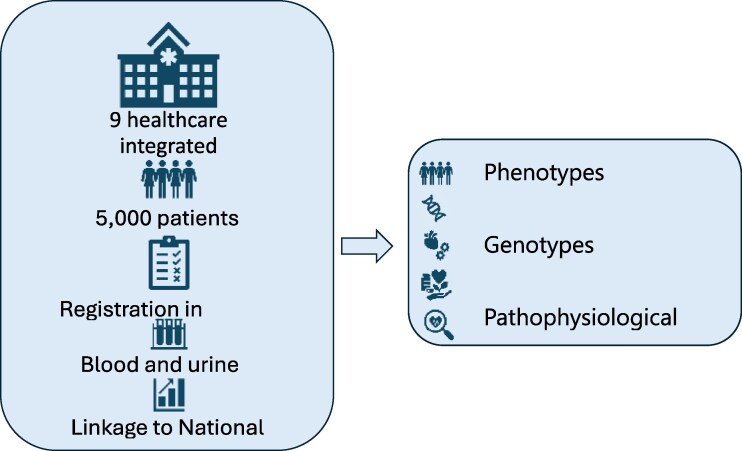
Study design of the SwedeHF Biobank

The Study design is outlined in *Figure 1* and the Graphical abstract. Nine hospitals registering HF patients in SwedeHF and with access to central biobanking in Sweden participate. Patients with HF are at registration in SwedeHF, and after signing the informed consent form, asked to undergo blood- and urine sampling (*Table 1*) including a laboratory screening panel at the local laboratory (*Table 2*) with an optional second sampling after 6 months. Samples are collected in adjunction to the acute hospitalization in those patients that are registered in SwedeHF during the index event or in stable condition in patients registered as out-patients. Inclusion and exclusion criteria are presented in *Table 3*. Enrolled patients are handled within routine clinical care according to guidelines with continuous registrations in SwedeHF. The study protocol is approved by the Swedish ethics review authority (218/443-31) and registered in ClinicalTrials.gov (NCT06435585).

**Table 1:** Blood and urine sampling in the SwedeHF Biobank

Blood	Aliquots	Storage
1 × 10 mL of blood in a serum tube	Divided into 8–16 aliquots	−70°C according to standardized routine at the laboratory.
3 × 10 mL EDTA tubes	Divided into 16–24 aliquots	−70°C according to standardized routine at the laboratory.
2 × 5 mL citrate tubes	Divided into 4–8 aliquots	−70°C within 2 h according to standardized routine at the laboratory.
2 × 10 mL EDTA tubes of whole blood for DNA extraction –	Divided into 4 cryo vials a 5 mL each	−70°C according to standardized routine at the laboratory.
Urine		
1 × 10 mL of urine	Divided into 4–16 aliquots	−70°C according to standardized routine at the laboratory.

Approximately 70 mL blood and 10 mL of urine will be collected at enrolment, and if possible, a second time at 6-month follow-up.

**Table 2:** Blood and urine sampling in the SwedeHF Biobank

Local laboratory screen
**NT-proBNP**
**Creatinine**
**Potassium**
**Sodium**
**ALT**
**TSH**
**AST**
**ALP**
**Bilirubin, total**
**Transferrin saturation**
**Ferritin**
**Haemoglobin**
**Platelet count**
**Leukocyte count**
**Leukocyte differential count**
**Glucose**
**HbA1c**
***Optional***
**Uric acid**
**hs-TNT**
**hsCRP**

Local laboratory screening will be performed at enrolment, and if possible, a second time at 6-month follow-up.

**Table 3:** Inclusion and exclusion criteria in the SwedeHF Biobank

**Patients with the diagnosis of HF registered in the SwedeHF registry at enrolling hospitals.** **Inclusion criteria** Written informed consent Heart failure defined by symptoms and signs of heart failure as judged by the local investigator Registered in SwedeHF **Exclusion criteria** Plasma donation within 1 month of enrolment or any blood donation/blood loss >500 mL during the 3 months prior to enrolment Previous allogeneic bone marrow transplant (genetics) In the opinion of the investigator, condition/s that may either put the patient at risk on participation or influence the results or the patient’s ability to participate in the study.

### The SwedeHF registry

Started in 2003, SwedeHF is the world’s largest continuous HF registry. The registry enrols patients with a clinical diagnosis of HF (regardless of LVEF) and after 2017 defined by ICD-10 codes I50, I420, I426-7, I255, I110, I130, and I132 at time of hospital or clinical visit https://www.ucr.uu.se/rikssvikt/. There are >200 000 registrations from >140 000 unique patients with ∼8 000 unique registrations added every year from 69 out of 76 hospitals in Sweden. Analyses from the registry have illustrated how HF care is conducted in a real-world HF population throughout Sweden and RRCTs have identified under treatment and facilitated implementation of GDMT resulting in improved quality of HF care.^15^ The SwedeHF registry consists of clinical information entered locally and then managed and stored by Uppsala Clinical Research Centre (UCR) according to national regulatory requirements. Approximately 80 variables are recorded including X-ray, ECG, heart rate, blood pressure, New York Heart Association class, comorbidities, cardiovascular treatments and the validated QoL instrument EQ-5D. The LVEF is the only echo parameter reported. Data is collected from SwedeHF at the timepoint of biomarker sampling and at the optional 6-month follow-up.

### Additional detailed clinical data

Study data specifically relating to the Biobank (primarily local lab panel; *Table 2*) not captured in SwedeHF will be collected locally and stored and managed in an electronic data capture (EDC) system using the REDCap (Research Electronic Data Capture) tools hosted at Karolinska Institutet. REDCap is a secure, web-based software platform designed to support data capture for research studies, providing (1) an intuitive interface for validated data capture; (2) audit trails for tracking data manipulation and export procedures; (3) automated export procedures for seamless data downloads to common statistical packages; and (4) procedures for data integration and interoperability with external sources.

Additional data on comorbidities, treatments and outcomes will be provided by linking SwedeHF with other government and disease registries through the unique personal identification number, including the Dispensed Drug Register, National Patient Register and National Cause of Death Register (all administered by Socialstyrelsen—The National Board of Health and Welfare)^16^ with repeated data extractions 4 times yearly.

### Biobank datasets

The results from future biomarker and genetic analyses will be linked with the described clinical datasets (SwedeHF, REDCap, and The National Board of Health and Welfare registries including outcomes). Data extractions will create a study dataset with appropriate patient selection and timepoints for the specific research project. The specific dataset will only be accessible coded for the researcher after project approval by the Biobank Steering Committee and Swedish Ethical Review Authority. All data will be strictly handled according to the EU Regulation 2016/679 (General Data Protection Regulation; GDPR).

### The Biobank

The Biobank builds on the structure of healthcare integrated biobanking in Sweden (https://biobanksverige.se/en/research/). This is a national model aiming to collect, handle, and store samples for research through routine sampling in healthcare, increasing patient participation within research involving biobank samples. In compliance with the Swedish Biobank Act (2023:38) all samples are labelled with unique bar codes and traceable to the donor and unique Swedish personal identification (ID) number held by all permanent residents in Sweden but without actual personal ID marked on the vials. This ensures a standardized handling of biomaterial for long-term storage with high security and traceability.

### Chain of custody of biological samples

Sampling, in total of 70 mL blood (serum, plasma, whole blood) and 10 mL urine, will be collected and stored in Biobank (*Table 1*). The biobanked samples will be stored in alarmed −70°C freezers at the local hospital laboratory in a systematic and qualitative manner.

### Circulating and urine biomarkers in HF

We will in plasma, serum, whole blood and urine analyze proteins and metabolites, as well as gene expression. We will identify responders and non-responders to treatment, explore molecular HF phenotypes, and identify disease mechanisms and potential novel drug targets.

Potential biomarkers indicative of various pathophysiological processes and disease mechanisms in HF are projected. These may include, but are not limited to, protein markers of congestion (natriuretic peptides), adrenomedullin, carbohydrate antigen 125, fibroblast growth factor 23,^17,18^ myocardial stress/damage (troponins, myoglobin, and copeptin),^19^ fibrosis (collagen markers as collagen I and II, PIICP, CITP, MMPs—MMP1, MMP9, and growth factors),^20^ inflammation and endothelial dysfunction (IL-6, IL-8, GDF-15, sST2, pentraxin, E-selectin, VCAM, endothelin, TGFβ, galactin-3 and cytokines),^8,21–23^ metabolic derangements (glucose, insulin, adiponectin, leptin, NRG1, apelin, IGF1 and IGFBP1-7) and mitochondrial dysfunction (micronutrients) and broader proteomic profiles.^24,25^ Urine biomarkers may include album, creatinine and kidney-specific urinary epidermal growth factor.^26^

Metabolites, i.e. small molecules of metabolic intermediates such as substrates and products of carbohydrate, amino acid, fatty acids, and ketone metabolism involved in cellular and organism homeostasis will be analyzed.^27^ Metabolic profiling may be used to explore pertinent systemic metabolic dysfunction in HF,^28^ distinguish specific HFpEF pathophysiology from HFrEF^29^ and identify activated pathways and add prognostic information.

### Genetics and transcriptomics

A discovery phase with array-based genotyping will identify common and low-frequency single-nucleotide gene variants that associate with HF subtypes and outcomes including mortality and disease progression. We will seek to validate identified gene variants in other cohorts.^30^ Previously identified target genes for HF and its subtypes^31^ will also be evaluated for clinical utility by association with outcomes and interaction with HF treatments. Large numbers of genetic variants of individually small effect will be combined into polygenic risk scores. This approach has the potential to elucidate polygenic and oligogenic contributions to HF outcomes and suggest guided therapy.

We will also explore the role of circulating microRNAs (miRNAs), which are single-stranded untranslated RNA molecules of 19–25 nucleotides in length that regulate gene expression by binding to the 3´-UTR in the mRNA of their target genes, thereby inhibiting the translation into protein or destabilizing the mRNA. Several circulating miRNAs have been identified as putative biomarkers for HF.^32^

### Sample size

The overall sample size calculation was based on results from the BIOSTAT-CHF (Biology study to tailored treatment in chronic heart failure) study. BIOSTAT-CHF had a similar systems biology approach as the SwedeHF Biobank but was more focused on mathematical simulations and genetic studies (GWAS and proteomics). BIOSTAT-CHF included 2516 severely diseased HFrEF patients^17^ whereas the SwedeHF Biobank aims to include non-selective HF patients across LVEF fractions, including less severely diseased individuals. Thus in order to fulfil all aims an estimation of 5000 enrolled patients is considered needed.

### Statistical methods—bioinformatics, machine learning models, network and pathway analyses

The proteomics, metabolomics, transcriptomics and genomics in circulating and urine biomarkers and patient characteristics data will be investigated in bioinformatic analyses; phenomapping models, machine learning and clustering models Principal Component Analysis (PCA), Projection of Latent Structures-Discriminant Analysis (PLS-DA) and Orthogonal projection to latent structures by partial least square (OPLS) by SIMCA software; Umetrics, Umeå, Sweden (http://umetrics.com). Traditional Cox regression and Mendelian randomization models will also be used.

Gene expression analyses will determine differentially expressed genes (DEGs). Bioinformatic information exploring upstream pathways, pathophysiological processes and network functional enrichment analyses will be performed by tools such as Gene Ontology (GO) resources, Kyoto Encyclopaedia of Genes and Genomes (KEGG).

### Current status

The Biobank had recruited 1348 patients between January 2021 and September 2024 and is currently continuing enrolment. Of recruited patients 469 (35%) have returned for optional second sampling after 6 months. Baseline characteristics of the 1348 patients are presented in *Table 4* including key variables from the SwedeHF registry during 2023. In the 1348 initially recruited patients in the Biobank study median age was 72 years, (quartile 1–3; 63–79) and there were 30% females compared with 75 (66–81) years and 36% females among the 8506 patients with an index registration in SwedeHF 2023.

**Table 4:** Baseline characteristics of patients enrolled in the SwedeHF Biobank and all patients with an index visit in SwedeHF during 2023

Variable	Biobank (*n* = 1348)		SwedeHF index 2023 (*n* = 8506)	
***Demographics***				
Sex, *n* (%)				
Female	403	(30%)	3069	(36%)
Male	945	(70%)	5437	(64%)
Age, years, median (Q1–Q3)	72	[63–79]	75	[66–81]
HF duration, *n* (%)				
<6 months	873	(72%)	5814	(70%)
≥6 months	334	(28%)	2494	(30%)
LVEF (%), *n* (%)				
HFpEF	146	(11%)	1655	(20%)
HFmrEF	325	(24%)	2263	(28%)
HFrEF	877	(65%)	4162	(52%)
NYHA class, *n* (%)				
I	176	(13%)	1120	(16%)
II	650	(48%)	3747	(52%)
III	402	(30%)	2264	(31%)
IV	10	(1%)	62	(1%)
*Medical history/comorbidities, n (%)*				
Previous myocardial infarction	302	(22%)	2038	(24%)
CABG	75	(6%)	445	(5%)
PCI	194	(14%)	1306	(16%)
Hypertension	791	(59%)	5225	(62%)
Atrial fibrillation/flutter	614	(46%)	4167	(49%)
Diabetes	315	(23%)	2016	(24%)
Chronic lung disease	169	(13%)	1217	(14%)
Heart valve disease	186	(14%)	1304	(16%)
Valve surgery	91	(7%)	603	(7%)
Aorta	58	(4%)	386	(5%)
Mitralis	35	(3%)	144	(2%)
Dilated cardiomyopathy	180	(13%)	464	(6%)
*Primary aetiology n (%)*				
Hypertension	225	(17%)	1495	(24%)
Ischemic heart disease	267	(20%)	1807	(29%)
Dilated cardiomyopathy	114	(8%)	278	(4%)
Known alcoholic cardiomyopathy	4	(0.5%)	42	(1%)
Heart valve disease	70	(5%)	462	(7%)
Other	292	(22%)	2166	(35%)
Systolic blood pressure (mmHg) median [Q1–Q3]	120	[110–135]	126	[112–140]
Diastolic blood pressure (mmHg) median [Q1–Q3]	75	[67–82]	75	[67–83]
Heart rate (beats/min) median [Q1–Q3]	70	[60–81]	72	[62–84]
*Laboratory measurements median (Q1–Q3)*				
NT-proBNP (pg/ml)	1240	[470–2830]	1560	[629–3617]
Creatinine (μmol/L)	90	[76–112]	90	[75–111]
Potassium (mmol/L)	4.2	[4.2–4.5]	4.0	[4.0–4.0]
Sodium (mmol/L)	140	[138–141]	140	[138–142]
Hemoglobin (g/L)	140	[128–151]	137	[124–149]
Ferritin (μg/L)	164	[79–302]	135	[65–270]
Transferrin (%)	25	[18–34]	23	[15–32]
*Treatments n (%)*				
RASi/ARNi HFrEF	808	(91%)	3954	(95%)
Beta-blocker HFrEF	1010	(93%)	3871	(93%)
MRA HFrEF	644	(73%)	2726	(66%)
SGLT2 inhibitor HFrEF	647	(77%)	3336	(81%)
SGLT2 inhibitor HFmrEF	182	(60%)	1455	(65%)
SGLT2 inhibitor HFpEF	77	(55%)	844	(51%)
ICD implanted LVEF ≤35% or <40%	59	(7%)	202	(5%)
CRT implanted ≤35% or <40% and QRS >130 ms	75	(6%)	126	(17%)

Categorical variables are presented as numbers and percentages, continuous variables as median and interquartile range.

AF, atrial fibrillation; ARNI, angiotensin receptor-neprilysin inhibitor; CABG, coronary artery bypass graft surgery; CRT, cardiac resynchronization therapy; HF, heart failure; HFmrEF, mildly reduced ejection fraction heart failure; HFpEF, preserved ejection fraction heart failure; HFrEF, reduced ejection fraction heart failure; HR, heart rate; ICD, implantable cardioverter defibrillator; Q1–Q3, quartile 1–3; LVEF, left ventricular ejection fraction; MRA, mineralocorticoid receptor antagonists; NT-proBNP, N-terminal prohormone B-type natriuretic peptide; NYHA, New York Heart Association; PCI, percutaneous coronary intervention; RASi, renin-angiotensin system inhibitors; SGLT2 inhibitor, sodium/glucose co-transporter 2 inhibitor.

A proportion of 11% of the Biobank participants had an LVEF ≥50% and 65% had an LVEF <40% compared with 52% and 20% respectively in patients with an index registration in SwedeHF 2023. The Biobank population exhibited functional NYHA class I in 13%, II in 48% and III in 30% and a burden of co-morbidities such as diabetes, hypertension (59%) and atrial ﬁbrillation (46%) corresponding to SwedeHF 2023. N-terminal prohormone of brain natriuretic peptide (NT-proBNP) was in the Biobank population 1240 [470–2830] vs 1560 [629–3617] pg/mL in SwedeHF. The use of GDMT therapy was similar. Of the Biobank participants compared with SwedeHF 91% vs 95% were treated with RASi/ARNi, 93% vs 93% with beta-blockers and 73% vs 66% with MRA (*Table 4*).

## Discussion

Combining biological data with detailed patient information, and outcomes will open for unique opportunities characterizing HF phenotypes. The use of modern large-scale proteomics, metabolomics, and genomics in combination with statistical modelling will provide excellent possibilities to identify novel biomarkers, uncover complex biological interactions, and reveal mechanistic insights that are not accessible through single-omics approaches. By integrating these high-dimensional datasets, researchers can improve disease classification, predict therapeutic responses and generate more accurate models of biological systems. Furthermore, this approach enables the discovery of subtle molecular patterns, supports personalized medicine initiatives and facilitates the translation of basic research findings into clinically relevant applications. Also, analyses may create more homogenous groups sharing pathophysiological mechanisms responding to existing treatment and/or identify new targets for intervention. Further the SwedeHF Biobank can be the foundation of simple pragmatic RRCTs enriched with molecular derived data.

### The SwedeHF Biobank population

The Biobank population consists of HF patients enrolled in the national quality registry SwedeHF. The initially enrolled patients have compared with the larger overall SwedeHF registry fairly similar characteristics, but with a higher proportion of HFrEF, 65% vs. 52% respectively. There are previous HF cohorts that have provided valuable proteomic and genomic information in HF such as the European BIOSTAT-CHF index cohort which recruited 2516 HF patients 2010–2014^33^ later amended by a comparable validation cohort (*n* = 1738) and the Asian Singapore Heart Failure Outcomes and Phenotypes (SHOP) which enrolled 2039 patients during 2010–2014^34^ (ACTRN12610000374066). In comparison the initial SwedeHF Biobank vs BIOSTAT-CHF, patients are older (76 vs. 69 years) with lower NT-proBNP (1240 vs. 4275 pg/mL) in line with a lower proportion of HFrEF (65% vs. 93% in the index cohort) respectively. Our HF population is projected to be larger, 5000 patients, on modern GDMT.

### Biomarkers reflecting pathophysiological mechanisms and benefit of treatment in HF—responders and non-responders

In HF natriuretic peptides (NPs), primarily brain (B-type) natriuretic peptide (BNP) or NT-proBNP, are used and well validated for diagnosis and risk assessment across ejection fractions.^35^ Even if lower concentrations of NT-proBNP are associated with reversed cardiac remodelling and better outcomes, using NT-proBNP to guide treatment in HFrEF has not reduced outcomes.^36^ However, there are patients responding with decreasing NPs after receiving GDMT. A reduction in NT-proBNP of >30% or concentrations <1000 pg/mL has been demonstrated associated with a lower risk of mortality and morbidity and reversed remodelling and suggested as a definition of responders to treatment.^35^ The question is who are the patients responding—or not responding—and why does treatment response differ?

Differences in response may be related to age, concomitant diseases or to structural changes of the myocardium. BNP reflects stretch of the myocardium, especially the ventricles, but increased concentrations may also be due to non-cardiac causes. Conditions such as inflammation, oxidative stress, fibrosis and myocardial necrosis are important factors in development and underlying pathophysiology of HF and biomarkers reflecting such conditions add to NT-proBNPs prognostic information^37^ or even prevail its prognostication.^8^ Biomarkers reflecting treatment response, relating to changes in the underlying pathophysiology^38^ provides us with druggable treatment targets.^39,40^

### Phenotyping

By analyzing a range of biomarkers, we see patterns shared within subgroups or phenotypes. There have been several reports exploring differences in protein or metabolite expression in HF phenotypes according to established characteristics such as LVEF^29,41^ and HF stages^42^ that may respond differently to treatment.^43^ In HFpEF we and others have through biomarkers provided information on inflammation, immune activation, endothelial dysfunction and pathophysiological pathways.^8,44^

There are differences across the LVEF spectrum but probably also shared pathological processes and drivers of poor HF prognosis.^40^ Novel, but also existing, treatments may be beneficial regardless of LVEF, more so in subgroups with shared underlying pathophysiology.

The role of genetic polymorphisms in HF development and outcomes and implication in treatment response has been suggested but so far with limited clinical utility. Still there are reports that genetic variants modulate the response to candesartan in HFrEF,^45^ spironolactone in HFpEF^46^ and furosemide-based diuretic regimen in patients with decompensated HF.^47^ Also, beta-blocker response varies,^48^ partly with polymorphism in the CYP2D6 gene regulating the predominant metabolizing enzyme of carvedilol and metoprolol.^49^ However, results diverge and need to be validated in order to be translated into clinical practice. We can provide large-scale genome-wide studies with adequate methodology and statistical analysis enabling genetic tailoring of HF therapy. In the future development of deep learning algorithms and artificial intelligence-powered reading can combine genetic and biomarker data and information from national registries to further identify HF phenotypes.^50^ In more system biology approach, integrating genetic, transcriptomic, and proteomic data in machine learning models novel pathways may pave the way for new treatment targets.^38^

### A platform for registry-based randomized clinical trials—RRCT

The concept of a RRCTs is a randomized controlled trial (RCT), but pragmatic and simple to perform, with efficient enrolment and less expensive compared with traditional RCTs. The structure of a Biobank within SwedeHF is a platform well suited for performing RRCTs, taking the concept to the next level including also biological information. Data collection and biobanking performed within clinical care in hospitals all over Sweden facilitates enrolling nationwide real-world populations of HF patients.

### Ongoing studies

There are several ongoing registries or study cohorts integrating clinical data with proteomic and genomic information in HF populations exploring phenotypes and pathophysiology (*Table 5*). Compared with the SwedeHF Biobank the majority are smaller studies from single centres or with focus on specific HF subgroups such as acute HF or HFpEF. All these studies will add to the knowledge in HF however none of them will collect contemporary clinical information from real-world patients in a national registry containing clinical data including information on cardiac function and molecular data with complete follow-up.

**Table 5:** Study cohorts with HF patients including biobanks

Study cohort	Population	*N*	Year	Outcome	Objective
Preserved versus Reduced Ejection Fraction Registry and Precision Medicine Database for Ambulatory Patients with Heart Failure (PREFER-HF; NCT03480633) (Abboud, 2021 #2910)	HF patients	3000	2016 2027	All-cause mortality and HF hospitalizations	To evaluate distinct sub-groups of HF (HF phenotypes) and cardiomyopathies including amyloidosis with an ultimate goal to optimize and individualize therapy with maximal benefits and minimized side effects. PREFER-HF recruits 3000 HF patients in a single centre study at Massachusetts General Hospital in Boston, US
Predicting Readmissions Using Omics, Biostatistical Evaluate and Artificial Intelligence (PROBE AI; NCT05028686)	Hospitalized HF patients	500	2024 2029	All-cause/CV mortality and HF/CV hospitalizations	To predict HF readmissions using omics, machine learning, patient reported outcomes, clinical data and other high-dimensional data sources in 500 in patients with HF in Canada.
The Heart Failure Precision Medicine Study (NCT04196842)	HF patients	100	2019 2026	All-cause mortality, hospitalization, Mechanical circulatory support device or heart transplant	To explore if multi-omics can identify HF profiles at risk of adverse outcomes and evaluates a telemonitoring intervention to optimize GDMT in 100 HF patients in the US.
Chronic Heart Failure—COngestion eValuation (CHF-COV; NCT05089149)	Chronic HF patients	200	2021 2029	All-cause mortality and HF hospitalization	To identify congestion markers (clinical, biological and ultrasound) associated with outcome, Nancy, France.
Chronic Heart Failure With Reduced Ejection Fraction—COngestion eValuation) (CHF-COVReduced; NCT05089162)	Chronic HF patients with LVEF <50%	200	2021 2029	All-cause mortality and HF hospitalizations, IV diuretics injection	To identify congestion markers (clinical, biological and ultrasound) associated with outcome, Nancy, France.
Acute Heart Failure With Preserved Ejection Fraction—COngestion Discharge Evaluation (AHF-CODE-P; NCT04343430)	Hospitalized HF patients	170	2020 2028	All-cause mortality and HF hospitalizations, IV diuretics injection	To identify congestion markers (clinical, biological and ultrasound) collected at the end of HF hospitalization associated with outcome, Nancy, France
Acute Heart Failure With Reduced Ejection Fraction—COngestion Discharge Evaluation (AHF-CODE-R; NCT04343443)	Hospitalized HF patients	200	2020 2028	All-cause mortality and HF hospitalizations, IV diuretics injection	To identify congestion markers (clinical, biological and ultrasound) collected at the end of HF hospitalization, Nancy, France
Acute Heart Failure—COngestion Repeated Evaluation (AHF-CORE; NCT03327532)	Hospitalized HF patients	80	2018 2026	All-cause mortality and HF hospitalizations, IV diuretics injection	To identify congestion markers (clinical, biological and ultrasound) collected at beginning and end of HF hospitalization associated with outcome, Nancy, France
Next-generation, Integrative, and Personalized Risk Assessment to Prevent Recurrent Heart Failure Events (ORACLE study; NCT05679713)	HF patients	1134	2022 2024	All-cause mortality and HF hospitalizations	To develop and validate an algorithm integrating blood RNA-based biomarkers, clinical, and patient-centred data and to assess the incremental predictive value compared with a traditional risk model, Barcelona Spain.
Cohort Study of Chronic Heart Failure (CHF) NCT05960890 *N* = 1000 China	HF patients	1000	2023 2026	CV mortality, HF hospitalizations and HF intervention	To explore phenotype, environmental exposure, non-invasive biomarkers, multiple omics data, intestinal microbiome, genome, metabolome, and association with outcome, applying remote monitoring assisted by community physicians, China.
A Registry Study of Biomarkers in Progression of Acute Heart Failure (BIOMS-POAHF; NCT04108182) (Ma, 2022 #3216)	HF patients	850	2015 2025	All-cause mortality and HF hospitalizations	To discover the prognostic value of biomarkers in acute heart failure, China
UK Heart Failure With Preserved Ejection Fraction (UK HFpEF; NCT05441839) (, 2024 #3214}	HFpEF patients	10 000	2022 2037	Identify subgroups of HFpEF	To develop a large, highly characterized cohort of patients with HFpEF recruited at 47 sites in UK. A biobank will be established. Deep clinical phenotyping, imaging, multi-omics and centrally held national electronic health record data will be integrated at scale, in order to reclassify HFpEF into distinct subgroups.
Prospective Registry of Acute Heart Failure (NCT02444416) {Carballo, 2020 #3217)	Hospitalized HF patients	1200	2014 2026	All-cause mortality	To create an observational registry of all HF hospitalized patients, explore aetiologies and prognostic factors, the specific role of acute renal failure, collection of whole blood for genetic analyses, single centre, Geneva Switzerland

## Limitations

Despite the SwedeHF is a generalizable, large Biobank recruiting patients from all over Sweden it has limitations. Recruitment is mainly performed at major hospital clinics that have integrated Biobank systems. This may result in patients recruited are more likely to be HFrEF patients as HFpEF patients often are managed in primary care. Also, the second blood sampling will not be performed by all patients limiting the opportunities for performing longitudinal studies.

## Conclusion

In the SwedeHF the world’s largest continuous HF registry, we have initiated a high-quality biobank, consisting of plasma, serum, whole blood and urine samples. The Biobank will enable future studies exploring underlying disease mechanisms in HF and response to HF treatment, paving the way for precision medicine and novel drug targets. The SwedeHF Biobank will provide unique opportunities for future research including pathophysiological aspects and offers a structure for biobanking in RRCTs within the national SwedeHF registry.

## Declarations

### Data Availability

No data were generated or analysed for this manuscript.
